# Chemolithoautotrophic arsenite oxidation by a thermophilic *Anoxybacillus flavithermus* strain TCC9-4 from a hot spring in Tengchong of Yunnan, China

**DOI:** 10.3389/fmicb.2015.00360

**Published:** 2015-05-06

**Authors:** Dawei Jiang, Ping Li, Zhou Jiang, Xinyue Dai, Rui Zhang, Yanhong Wang, Qinghai Guo, Yanxin Wang

**Affiliations:** ^1^State Key Laboratory of Biogeology and Environmental Geology, China University of GeosciencesWuhan, China; ^2^School of Environmental Studies, China University of GeosciencesWuhan, China

**Keywords:** arsenite oxidation, *Anoxybacillus*, temperature, hot springs, Tengchong

## Abstract

A new facultative chemolithoautotrophic arsenite (As^III^)-oxidizing bacterium TCC9-4 was isolated from a hot spring microbial mat in Tengchong of Yunnan, China. This strain could grow with As^III^ as an energy source, CO_2_–HCO_3_^-^ as a carbon source and oxygen as the electron acceptor in a minimal salts medium. Under chemolithoautotrophic conditions, more than 90% of 100 mg/L As^III^ could be oxidized by the strain TCC9-4 in 36 h. Temperature was an important environmental factor that strongly influenced the As^III^ oxidation rate and As^III^ oxidase (Aio) activity; the highest Aio activity was found at the temperature of 40∘C. Addition of 0.01% yeast extract enhanced the growth significantly, but delayed the As^III^ oxidation. On the basis of 16S rRNA phylogenetic sequence analysis, strain TCC9-4 was identified as *Anoxybacillus flavithermus*. To our best knowledge, this is the first report of arsenic (As) oxidation by *A. flavithermus*. The Aio gene in TCC9-4 might be quite novel relative to currently known gene sequences. The results of this study expand our current understanding of microbially mediated As oxidation in hot springs.

## Introduction

Geothermal waters often contain high levels of arsenic (As) and may contribute to surface and ground water contaminations (Langner et al., 2001; Zhang et al., 2008). As the waters equilibrate with the oxygen in outflow channels, arsenite (As^III^) is oxidized via microbial processes (Wilkie and Hering, 1998; Langner et al., 2001). To date, numerous As^III^ oxidizing mircrobes have been isolated from geothermal, sewage, soil, and mining environments.

Phylogenetically, As^III^ oxidizing microorganisms are often delineated according to different environmental temperature. For example, in geothermal environments, As^III^ oxidizers are found in bacterial phyla *Aquificae*, *Chloroflexi*, *Deinococcus–Thermus*, and in the *Archaea* (Mikael Sehlin, 1992; Gihring et al., 2001; Donahoe-Christiansen et al., 2004; Tang et al., 2011), while in mesophilic environments and low-temperature environments (<10°C), As^III^ oxidizers mainly belong to the *Proteobacteria* (Santini et al., 2000; Kashyap et al., 2006; Duquesne et al., 2008; Osborne et al., 2010). As a molecular marker, *aioA* has been used to investigate population structure and diversity of As^III^-oxidizing bacteria in a variety of As-contaminated environments (Inskeep et al., 2007; Hamamura et al., 2009; Osborne et al., 2010; Jiang et al., 2014).

Tengchong is a typical volcanic geothermal area located in southwestern China (Guo, 2012). It has more than 50 volcanoes and 140 geothermal fields (Du et al., 2005), with the largest two hydrothermal fields being located at Rehai and Ruidian (Guo and Wang, 2012). High concentrations of As have been found in hot spring waters (up to 687 μg/L), sediments and sinters in these geothermal areas (Zhang et al., 2008). Previous studies showed that diverse microbial communities inhabited these thermal environments (Hou et al., 2013). Our recent study of *aioA* diversity implied that novel lineages of microbial As oxidizers might inhabit this area (Jiang et al., 2014); however, microbially mediated As oxidation in this geothermal area has yet to be fully understood. The current study describes a new facultative chemolithoautotrophic As^III^-oxidizing bacterium isolated from a hot spring mat from the Rehai hot spring features, expanding our understanding of microbially mediated As oxidation in hot spring environments.

## Materials and Methods

### Site Description and Sample Collection

The Tengchong geothermal area is located in Yunnan province in southwestern China. It lies at the eastern end of the Yunnan–Tibet geothermal belt, a major part of the Mediterranean-Himalayas geothermal belt (Guo and Wang, 2012). Quaternary volcanoes and faults are the most conspicuous geologic features in this area (Du et al., 2005; Shangguan et al., 2005). Rehai is one of the largest geothermal fields within Tengchong, with large numbers of hot springs (Guo and Wang, 2012). A hot spring referred to as SRBZ (N24.9500°, E98.4375°) is located in the Rehai and is documented to have significant As loads; containing a total As concentration of 226.93 μg/L and arsenate concentration of 217.07 μg/L (Jiang et al., 2014). Microbial biomass in water was collected via filtration (0.22 μm × 142 mm diameter, Millipore Corporation, Billerica, MA, USA) and mat sample was collected with sterile spoons. All samples were placed into 50 mL sterile polypropylene tubes for transport to the lab.

### Isolation

For enrichment and isolation, minimal salt medium (MSM) was used. It contained (g/L): Na_2_SO_4_, 0.031; KH_2_PO_4_, 0.17; KCl, 0.15; MgCl_2_⋅6H_2_O, 0.04; CaCl_2_⋅2H_2_O, 0.05; (NH_4_)_2_SO_4_, 0.4; NaHCO_3_, 1.68; and trace elements and vitamins were referenced from Rhine et al. (2006). The pH of the medium was adjusted to 8.1 matching that of the SRBZ hot spring water.

About 1.0 g mat samples were incubated in 10 ml MSM containing 1.33 mM (100 mg/L) NaAsO_2_ (MSM-As) for 1 week at 40°C. Following four weekly 10-fold dilution transfers, the microbial suspension was diluted and spread onto solid plates with MSM-As agar (1.5%) plates amended with 0.1% yeast extract. After 2 days incubation, a number of different colonies were sub-cultured to purify and screen for As^III^ oxidization activity. Concentrations of As^III^ and arsenate (As^V^) were determined by using liquid chromatography–hydride generation–atomic fluorescence spectrometry (LC–HG–AFS, Haiguang AFS-9780, Beijing; Keller et al., 2014).

### Physiological and Biochemical Characteristics

Physiological and biochemical tests were carried out according to Pikuta et al. (2000). The effects of oxygen, temperature, pH, and NaCl optima were determined in MSM containing 0.01% yeast extract (MSM-YE). When potential carbon sources and inorganic substrates were tested, the strain was cultured in MSM, and the concentrations of carbon sources and inorganic substrates added to the medium were 10 mM. The anaerobic growth was observed in MSM-YE which was incubated in a Hungate tube at 60°C for 2 days.

Colony morphology was determined visually and microscopic characteristics were observed under the optical microscope (Leica DM5000 B). Cells of TCC9-4 were sent to Wuhan Institute of Virology, Chinese Academy of Sciences (CAS) after pre-culture with Luria–Bertani (LB) solid medium for negative-stain transmission electron microscopy (TEM, Hitachi H-7000FA) detection.

### DNA Extraction and Amplification of 16S rRNA and As^III^ Oxidase Genes

One isolate was obtained and was referred to as TCC9-4. DNA was extracted from over-night cultures or from the mat and the biomass-containing filter sampling from the SRBZ using FastDNA®SPIN Kit for Soil (Qbiogene, Inc. Carlsbad, CA, USA) according to the manufacturer’s protocols. DNA yields were quantified with a NanoDrop 2000 spectrophotometer (Thermo Scientific, USA). All PCRs were undertaken in Master cycler ep Gradient S Thermal Cycler block (Eppendorf, Hamburg, Germany). 16S rRNA gene was amplified with the primers P1 (5′ AGAGTTTGATCCTGGCTCAG 3′) and P2 (5′ GGTTACCTTGTTACGACTT 3′; Weisburg et al., 1991). A typical 50 μL PCR mixture contained 0.4 pmol/L of primers (MWG), 1 μL DNA template (50 ng), 10x reaction buffer (Promega Corporation), 5 mM MgCl_2_, 1.5 U Biotaq DNA polymerase (Promega), 0.25 m dNTP (Promega).

Several sets of PCR primers and conditions were employed to characterize the As^III^ oxidase (Aio) genes in the geothermal water and mat samples, and in an attempt to characterize the Aio gene of TCC9-4. The *aioA* gene of environmental samples was amplified with degenerate primers aroA95f (5′ TGYCABTWCTGCAIYGYIGG 3′) and aroA599r (5′ TCDGARTTGTASGCIGGICKRTT 3′; Hamamura et al., 2009) in a 25 μL PCR mixture consisting of PCR Ex Taq buffer, 100 μmol dNTP mixtures, 0.5 μmol primers, 50 ng templates, and 1 U of Ex Taq DNA polymerase (TaKaRa, Japan). The PCR program was as follows: initial denaturation at 94°C for 5 min, followed by nine cycles of denaturation at 94°C for 45 s, annealing at 54°C for 45 s, extension at 72°C for 1.5 min, and 25 cycles of denaturation at 94°C for 45 s, annealing at 50°C for 45 s, extension at 72°C for 1.5 min, and then a final extension step at 72°C for 7 min (Hamamura et al., 2009). Aio genes of TCC9-4 were amplified with seven pairs of degenerate primers, respectively, including aroA95f/aroA599r (Hamamura et al., 2009), set #1F/R, set #2F/R (Inskeep et al., 2007), aoxBM1-2F/aoxBM3-2R (Quemeneur et al., 2008), aoxFor/aoxRev (Clingenpeel et al., 2009), arxA_Deg_FB/arxA_Deg_RB, AOX-F-A2/AOX-R-E2 (Zargar et al., 2012). The PCR programs of Aio genes of TCC9-4 were referenced from these studies mentioned above.

### Clone Library Construction and Phylogenetic Analysis

PCR products were purified using the E.Z.N.A. Gel Extraction Kit (Omega Bio-tek, Inc. Norcross, GA, USA) according to manufacturer’s instructions. The purified PCR products were ligated into pMD-18T vectors (TaKaRa) and transformed into *Escherichia coli* DH5α competent cells. The transformed cells were plated on LB agar containing 100 μg/mL of ampicillin, 80 μg/mL of 5-bromo-4-chloro-3-indolyl-β-D-galactopyranoside (X-Gal) and 0.5 mmol isopropyl-β-D-thiogalactopyranoside (IPTG), and incubated overnight at 37°C. Randomly selected clones were commercially sequenced. The 16S rRNA gene sequence was aligned with closely related sequences in the GenBank database using the BLAST program. *aioA* gene sequences were edited in MEGA 5.05 and binned into various operational taxonomic units (OTUs) using DOTUR 1.53. One representative sequence from each OTU (0.03 cutoff) was selected for phylogenetic analysis. Prior to phylogenetic analysis, representative *aioA* sequences were translated into amino acid sequences and searched for closely related *aioA* amino acid sequences in the GenBank database (BLASTX), which were then used to construct a phylogenetic tree with MEGA 5.05. The 16S rRNA fragment nucleotide sequence of TCC9-4 has been deposited in the GenBank database under accession numbers KP027949. The *aioA* gene fragment nucleotide sequences obtained have been deposited in the GenBank database under accession numbers KC777327–KC777338.

### Chemolithoautotrophic and Heterotrophic Experiments

TCC9-4 was cultured in MSM-As for the chemolithoautotrophic growth and As^III^ oxidization experiments or MSM-YE for experiments examining heterotrophic growth. Cells were washed twice with distilled water and then inoculated (1% inoculum), and samples were taken periodically for the detection of cell growth and As speciation. Cell growth was measured by plate cell counting or optical density at 600 nm (OD_600_). Experiments were run in triplicate and duplicate, respectively.

### As^III^ Oxidase Assays

As^III^ oxidase activity was assayed as previously described by Anderson et al. (1992). Briefly, TCC9-4 was inoculated in 13 liters MSM-As and incubated for 48 h. Cells were harvested by vacuum filtration, washed three times with 20 mM Tris-HC1, 0.1 mM PMSF, 0.6 mM EDTA, pH 8.4, containing 0.9% NaCl, suspended and incubated in 20 mM Tris-HC1, 0.6 mM PMSF, 0.6 mM EDTA, pH 8.4, containing lysozyme (0.25 mg/ml), and then sonicated. Unbroken cells were removed by centrifugation, and then the crude extract was diluted with 50 mM 2-[*N*-morpholino]ethanesulfonic acid (MES) buffer (pH 6.0). Aio activity was determined by measuring the reduction of 2,4-dichlorophenolindophenol (DCIP) at OD_600_ in 50 mM MES containing 200 μM sodium As^III^. The blank was 50 mM MES buffer containing 120 μM DCIP and 200 μM sodium As^III^. The enzyme activity was defined as μmol of DCIP reduced/min/mg of protein. Protein concentrations were determined by Modified Bradford Protein Assay Kit (Sangon) using BSA as a standard according to manufacturer’s instructions.

## Results and Discussion

### Identification

**FIGURE 1:**
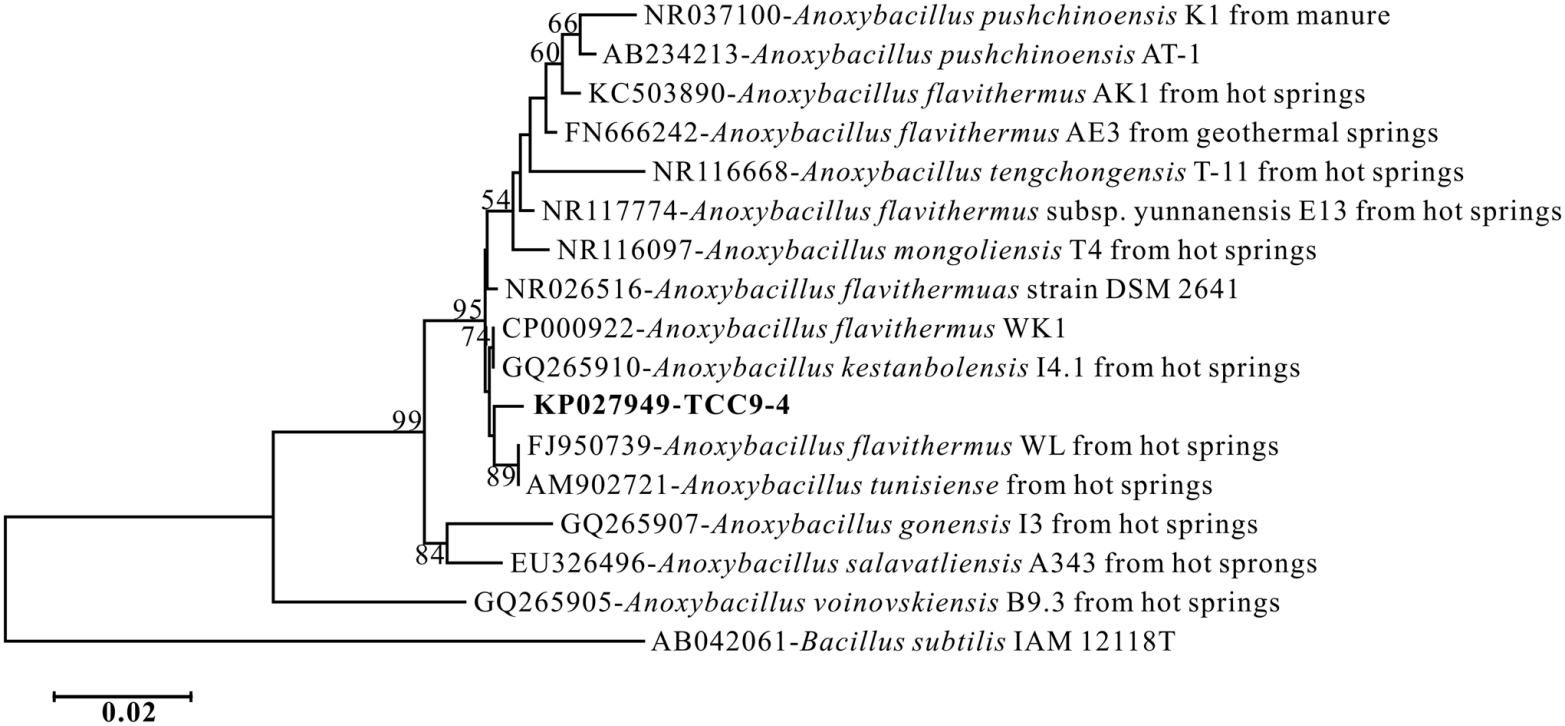
**Neighbor-joining tree showing the phylogenetic relationship of TCC9-4 belonging to the *Anoxybacillus* species.** The sequence of *Bacillus subtilis* was used as the outgroup. Significant bootstrap values from 1000 analyses are shown at the branch points of the trees. Only bootstrap values above 50% are shown. The GenBank accession number for each reference strain is shown before the strain name.

**FIGURE 2:**
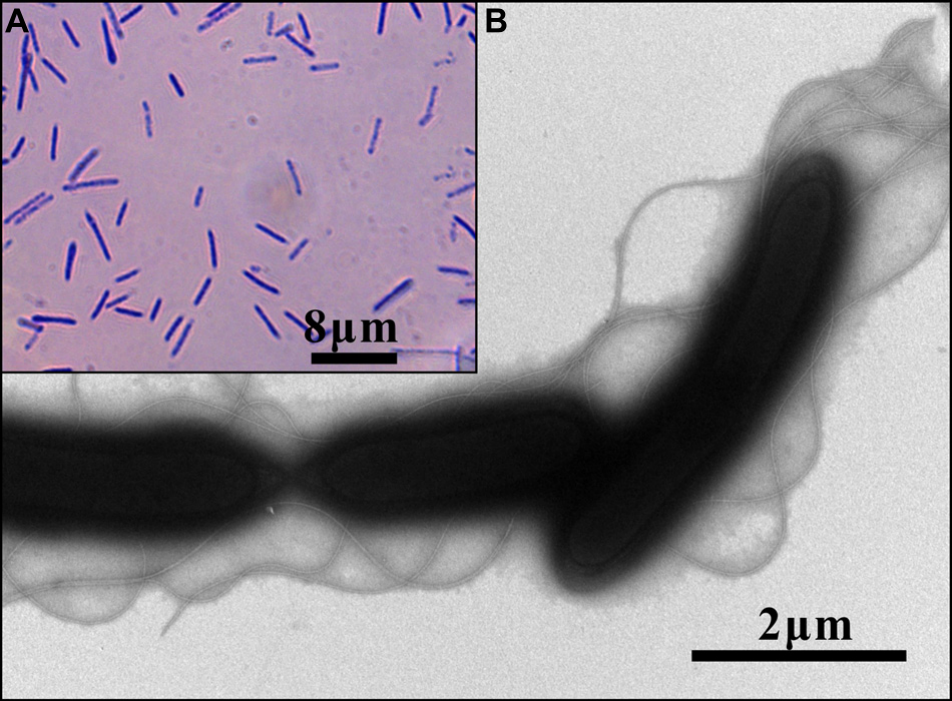
**Micrographs of strain TCC9-4. (A)** Light micrograph of TCC9-4 incubated in MSM-YE at 60°C. **(B)** Negative-stain transmission electron micrograph of TCC9-4 incubated in LB agar at 60°C.

A 1465 bp 16S rRNA gene sequence was obtained from PCR. BLAST analysis in GenBank identified that TCC9-4 belonged to the genus *Anoxybacillus* (99.66%). Phylogenetic analysis places TCC9-4 closest to two organisms referred to as *A. flavithermus* WL and *A. tunisiense* isolated in China and Tunisia, respectively (**Figure 1**). Phenotypic analysis found TCC9-4 to be a gram-positive, facultatively aerobic bacterium with a straight rod morphology and peritrichous flagella (**Figure 2**). The main physiological and biochemical characteristics of this isolate were investigated and compared with related species (**Table 1**). TCC9-4 has a pH range of 6.0–10.0 (optimum at 8.0), and is positive for methyl red test and nitrate reduction, but negative for gelatin hydrolysis, starch hydrolysis, tyrosine hydrolysis, and lysozyme tests. Growth was inhibited at NaCl concentrations higher than 3.0% (w/v). Utilizable carbon sources for heterotrophic growth include: maltose, sucrose, glucose, galactose, mannose, xylose, sodium citrate, tyrosine, mannitol, sorbitol, sodium acetate, sodium succinate, sodium formate, glycerin, and sodium lactate. Fructose, sodium oxalate, and inorganic substrates including Fe^II^, S^0^, and S^2-^ will not support growth. TCC9-4 is capable of growing in a chemolithotrophic mode using As^III^ as an energy source and CO_2_–HCO_3_^-^ as carbon source with oxygen as the electron acceptor (see below). The mol% G + C content of DNA is 56.63%. Based on 16S rRNA gene sequence and the above phenotype and biochemical characteristics, we conclude that TCC9-4 represents a new strain of *A. flavithermus.*

**Table 1:** Physiological and biochemical characteristics of TCC9-4 and related *Anoxybacillus* species.

Characteristics	TCC9-4	Species^a^							
		1	2	3	4	5	6	7	8
Oxygen requirement	FA	FA	FA	A	FA	ND	ND	ND	ND
Motility	+	+	+	-	+	ND	ND	ND	+
Temperature (∘C) range	30–68	30–65	35–75	37–66	37–69	30–70	30–70	30–70	30–66
Temperature (∘C) optimum	60	55	65	62	60	60	60	60	60
pH range	6.0–10.0	5.5–9.0	6.0–10.0	8.0–10.5	5.5–9.5	5.5–9.0	4.5–9.5	5.5–9.5	5.5–10.0
Tolerance to NaCl (%)	3.0	2.5	3.0	3.0	4.5	8.0	8.0	8.0	3.0
**Utilization of**									
Starch	-	-	+	+	+	+	-	-	-
Peptone	+	+	ND	-	ND	ND	ND	ND	+
Gelatin	-	-	ND	-	+	+	+	+	-
Yeast extract	+	+	ND	-	ND	ND	ND	ND	+
**Substrates utilized**									
D-Xylose	+	-	+	-	-	+	-	+	+
D-Galactose	+	+	+	-	+	+	-	+	+
D-Glucose	+	+	+	+	+	+	+	+	+
D-Fructose	-	ND	+	+	+	+	+	+	-
D-Mannose	+	+	+	ND	+	-	+	-	+
D-Maltose	-	ND	ND	ND	+	+	+	+	-
D-Mannitol	+	+	ND	ND	+	+	+	-	+
D-Sorbitol	+	ND	ND	-	-	-	+	-	ND
Sucrose	+	-	+	+	+	+	+	+	+
Lactate	+	-	ND	-	ND	ND	ND	ND	ND
Citrate	+	ND	ND	ND	-	-	-	-	ND
Glycerol	+	ND	ND	-	ND	+	+	+	ND
Nitrate reduction	+	+	ND	+	+	-	+	-	-
Methyl red test	+	+	ND	ND	-	ND	ND	ND	ND
Hydrolysis of tyrosine	-	-	ND	ND	-	ND	ND	ND	ND
Lysozyme present	-	ND	ND	ND	+	-	+	-	ND

ND, not determined; FA, facultative anaerobic; A, anaerobic; +, positive; -, negative.

^a^Species were as follows: 1: *Anoxybacillus flavithermus* DSM 2641T (formerly ‘*Bacillus flavithermus*’; Heinen et al., 1982); 2: *A. flavithermus* TWXYL3 (Ellis and Magnuson, 2012); 3: *A. pushchinensis* K1T (Pikuta et al., 2000); 4: *A. salavatliensis* A343T (Cihan et al., 2011); 5: *A. gonensis* I3 (İnan et al., 2011); 6: *A. voinovskiensis* B9.3 (İnan et al., 2011); 7: *A. voinovskiensis* I4.2 (İnan et al., 2011); 8: *A. flavithermus* E13T (Wang et al., 2014).

### As^III^ Oxidization

**FIGURE 3:**
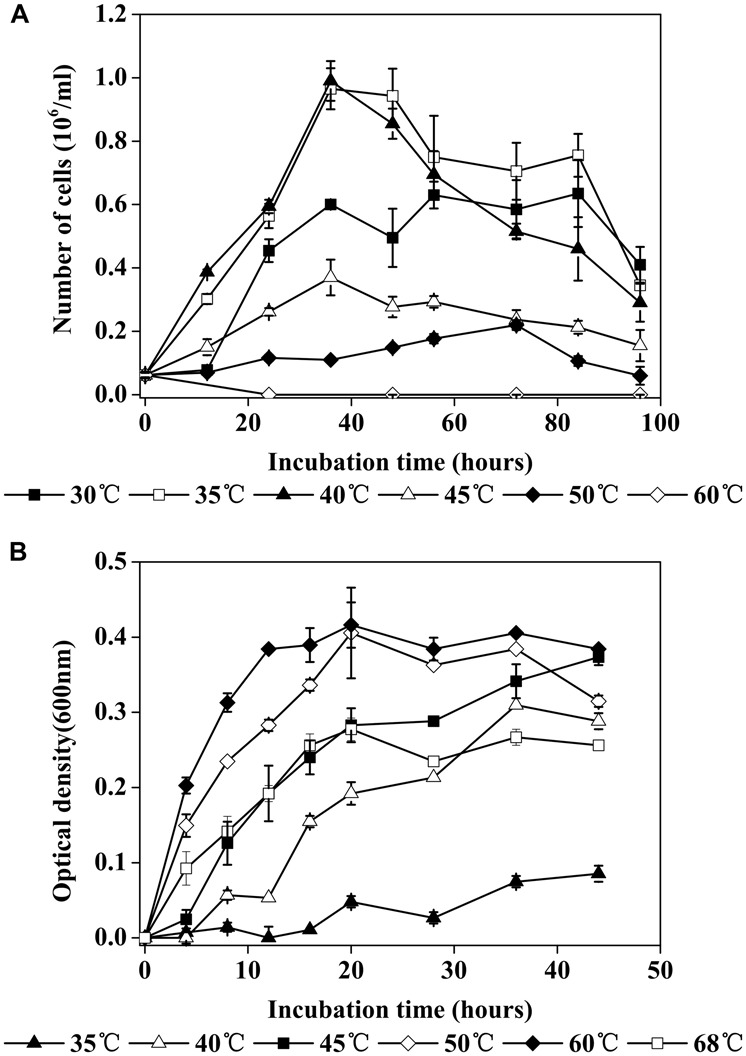
**(A)** Cell growth of TCC9-4 at different temperatures under chemolithoautotrophic conditions in MSM-As. Arsenite (As^III^) was the sole electron donor and CO_2_–HCO_3_^-^ was the carbon source for growth. Error bars are the SD of triplicate values. **(B)** Cell growth of TCC9-4 at different temperatures under heterotrophic conditions in MSM-YE. Error bars are the SD of duplicate values.

**FIGURE 4:**
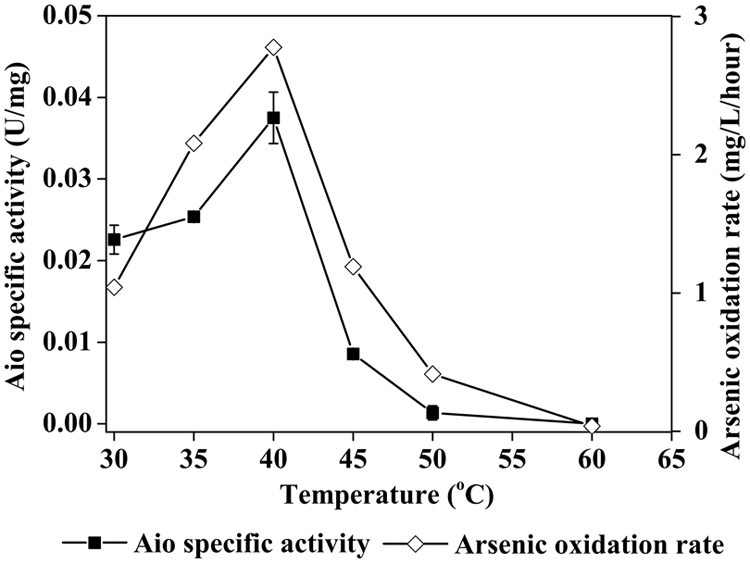
**Average As^**III**^ oxidation rates (*open diamonds*) and arsenite oxidase (Aio) activity (*solid square*) of strain TCC9-4 at different temperatures under chemolithoautotrophic conditions.** Error bars are the SD of triplicate values.

TCC9-4 temperature response varied as a function of mode of metabolism. When cultured chemolithotrophically with As^III^ as an electron donor in MSM-As, the optimal temperature was around 40°C (**Figure 3A**). The generation times of TCC9-4 at 30°C, 35°C, 40°C, 45°C, 50°C were 11.0 h, 9.1 h, 9.0 h, 14.0 h, 43.5 h, respectively. However, in MSM-YE, the temperature growth range was 30–68°C, with an optimum at 60°C (**Figure 3B**). Subsequent experiments then examined As^III^ oxidation in culture and crude cell lysates as a function of temperature (**Figure 4**). Optimum activity for both types of assays was 40°C. In whole cell culture assays at this temperature, more than 90% of As^III^ could be oxidized in 36 h and the highest As^III^ oxidization rate was 2.78 mg As^III^ oxidized/L/hour. The highest Aio activity was 0.037 U/mg at 40°C, matching cell growth behavior (**Figure 3A**) and suggesting the Aio enzyme temperature range is at least one critical limiting factor that constrains this organism in nature when growing in a chemolithotrophic mode. DCIP reduction was not detected in the blank without crude cell extract.

**FIGURE 5:**
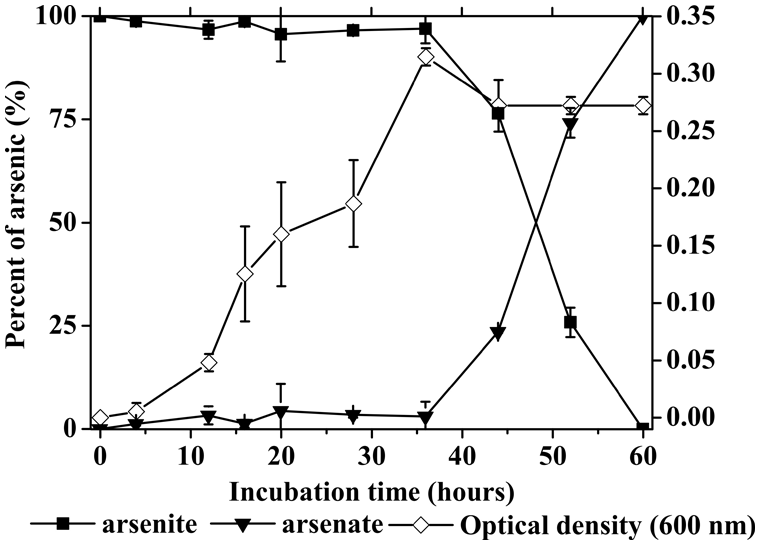
**As^**III**^ oxidation and cell growth of TCC9-4 at 40°C in MSM-YE-As.** The initial As^III^ concentrations were 100 mg/L. Error bars are the SD of duplicate values.

Additional experiments then examined if TCC9-4 could grow in both modes simultaneously. In MSM containing 0.01% yeast extract and 100 mg/L As^III^ (MSM-YE-As), As^III^ oxidation did not commence until the cells attained stationary phase (**Figure 5**), presumably due to the cells having already consumed all of the growth yielding substrates of yeast extract. We note, however, that As^III^ oxidation at this stage failed to yield growth benefits as no further cell growth was observed. This may was resulted from key inorganic nutrients (e.g., P, K, Mg, etc) being consumed and thus an additional energy source was perhaps not useful at this point. Also apparent from these studies, TCC9-4 clearly preferred heterotrophic growth on yeast extract. This behavior deviates from that observed in previous studies with *Rhizobium* sp. NT-26 and *Sinorhizobium* sp. KGO-5, where additions of yeast extract enhanced growth and accelerated As^III^ oxidizing significantly (Santini et al., 2000; Dong et al., 2014). Although the cell growth was enhanced due to the relatively higher optical densities, no obvious As^III^ oxidization has been observed during the logarithmic phase (**Figure 5**).

### As^III^ Oxidase Genes

**FIGURE 6:**
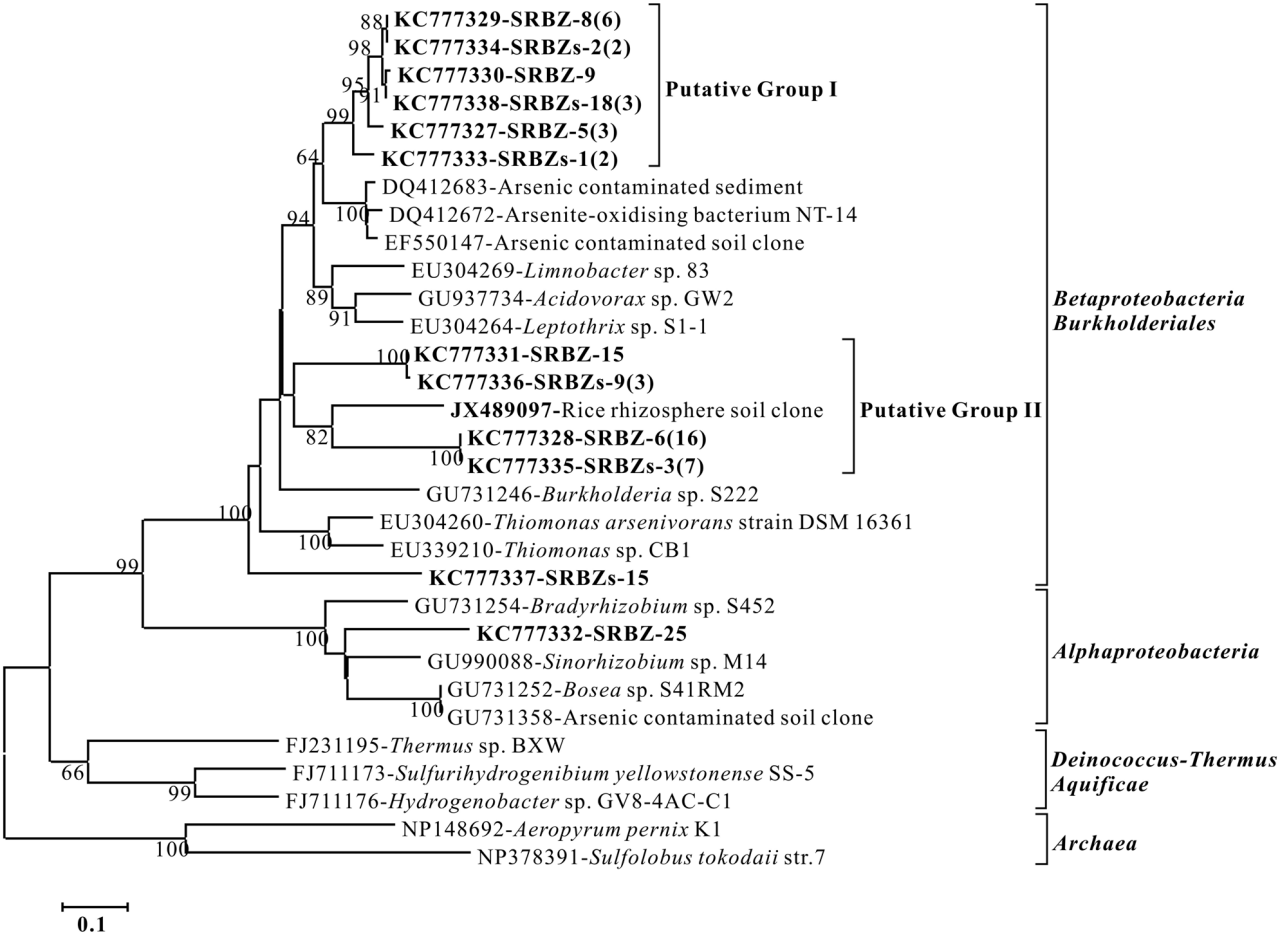
**Phylogeny of *aioA* sequences (165 unambiguous amino acids) deduced from clone sequences detected in the SRBZ hot spring.** Numbers in parentheses indicates the number of sequences obtained for each sequence type. Significant bootstrap values (per 100 trials) of major branch points are shown. Closely related groups of sequences have been designated putative group I and II.

We next sought to clone and characterize the Aio genes in this organism. To our knowledge, the apparent temperature sensitivity of this enzyme has not been reported previously and so it was of interest to attempt to understand potential structural features that might be associated with temperature sensitivity. All attempts to PCR amplification these genes failed. This included all known primers that span the known genes associated with As^III^ oxidation: genes referred to as *aro* (Hamamura et al., 2009); *aox* (Quemeneur et al., 2008); *arx* (Zargar et al., 2012). Failure to obtain a PCR product was not due to template issues because the 16S rRNA gene readily amplified. Rather, it would appear that the gene encoding the Aio in this organism is sufficiently different so that even highly degenerate primers failed to recognize this gene. Moreover, all available genome sequences of *A. flavithermus* including strain 25, WK1, AK1, TNO-09.006, E13, and Kn10 with GenBank assembly accession nos. GCA_000753775, GCA_000019045, GCA_000353425, GCA_000327465, GCA_000753835, and GCA_000367505, respectively, from GenBank have been checked. No *aioA* gene sequence could be identified in these genomes. However, the primers designed to amplify *aioA* (previously referred to as *aro*, *aox*, and *aso*) robustly generated PCR products when used with DNA extracted from the hot spring sediments as template. An *aioA* gene clone library of the mat was constructed and a total of 46 *aioA* gene clone sequences were obtained (**Figure 6**). Those *aioA* gene sequences were mainly classified into two putative groups and were closely related (72–80% amino acid level) to the Aio protein sequences from unidentified pure culture or gene clones of the order *Burkholderiales* of *Betaproteobacteria*.

As^III^-oxidizing bacteria have been isolated from geothermal, sewage, soil, and mine environments, including both heterotrophic As^III^ oxidizers and chemolithoautotrophic As^III^ oxidizers. Investigations of the As^III^ oxidation genes *aioA* indicated that As^III^ oxidizers have a widespread distribution in 11 phyla (Heinrich-Salmeron et al., 2011). And Heinrich-Salmeron et al. (2011) also reported As^III^ oxidizer–*Bacillus* sp. 21AAIII, expanding known As^III^ oxidation to the phylum *Firmicutes*. In this study, As^III^ oxidation in strain TCC9-4 further expands this trait in the *Firmicutes* exhibiting the capacity to grow chemolithotrophically with As^III^ and CO_2_. The carbon/energy substrates that can support the heterotrophic growth and phenotype characteristics of TCC4-9 were consistent with those reported for *A. flavithermus* DSM 2641T (Heinen et al., 1982). However, there are no reports of As oxidation in the genus *Anoxybacillus*. Moreover, no PCR product of *aioA* gene was obtained, nor could *aioA* be identified in any of the six available genome sequences of *A. flavithermus* in GenBank. Furthermore, no *aioA* gene has been found related to *Firmicutes* in the *aioA* gene clone library of the environmental sample from which strain TCC9-4 was isolated. This implies the Aio gene in TCC9-4 either has sufficiently low homology to currently known gene sequences so as to escape detection via PCR, or that this particular gene is quite novel relative to known enzymes catalyzing this reaction. Further studies that focus on the genes involved in As^III^ oxidation with genome sequencing of TCC9-4 and metagenomes sequencing of the source spring sample are warranted.

## Conflict of Interest Statement

The authors declare that the research was conducted in the absence of any commercial or financial relationships that could be construed as a potential conflict of interest.
